# Engineering of nanoparticle size via electrohydrodynamic jetting

**DOI:** 10.1002/btm2.10010

**Published:** 2016-06-20

**Authors:** Sahar Rahmani, Sumaira Ashraf, Raimo Hartmann, Acacia F. Dishman, Mikhail V. Zyuzin, Chris K. J. Yu, Wolfgang J. Parak, Joerg Lahann

**Affiliations:** ^1^ Biointerfaces Institute, University of Michigan Ann Arbor MI 48109; ^2^ Biomedical Engineering University of Michigan Ann Arbor MI 48109; ^3^ Institute of Functional Interfaces (IFG), Karlsruhe Institute of Technology (KIT) Karlsruhe Germany; ^4^ Dept. of Physics Philipps University of Marburg Marburg Germany; ^5^ Chemical Engineering University of Michigan Ann Arbor MI 48109

**Keywords:** biomaterials, cellular uptake, drug delivery, electrospraying, nanoparticles, size distribution

## Abstract

Engineering the physical properties of particles, especially their size, is an important parameter in the fabrication of successful carrier systems for the delivery of therapeutics. Here, various routes were explored for the fabrication of particles in the nanosize regime. It was demonstrated that the use of a charged species and/or solvent with high dielectric constant can influence the size and distribution of particles, with the charged species having a greater effect on the size of the particles and the solvent a greater effect on the distribution of the particles. In addition to the fabrication of nanoparticles, their fractionation into specific size ranges using centrifugation was also investigated. The in vitro particle uptake and intracellular transport of these nanoparticles was studied as a function of size and incubation period. The highest level of intralysosomal localization was observed for the smallest nanoparticle group (average of 174 nm), followed by the groups with increasing sizes (averages of 378 and 575 nm), most likely due to the faster endosomal uptake of smaller particles. In addition, the internalization of nanoparticle clusters and number of nanoparticles per cell increased with longer incubation periods. This work establishes a technological approach to compartmentalized nanoparticles with defined sizes. This is especially important as relatively subtle differences in size can modulate cell uptake and determine intercellular fate. Future work will need to address the role of specific targeting ligands on cellular uptake and intracellular transport of compartmentalized nanoparticles.

## 1 Introduction

During the last decades, the use of carrier systems for the delivery of therapeutics to specific locations in the body has gained significant momentum. Nanoparticle‐based delivery systems have evolved from simple, nontargeted drug carriers to highly multifunctional systems with various targeting, stealth, and delivery capabilities.^1^, ^2^, ^3^ There is evidence that the physical properties of a carrier system, such as size and shape, are as effective in determining the fate of the particles in the body as the chemical properties of the particles, such as targeting and stealth ligands incorporated on their surfaces.^4^, ^5^ Specifically, the size of a carrier system can influence the therapeutic loading percentages, the mode of delivery, induced toxicity levels, the circulation time in the body, the carrier's biodistribution in distinct organs, cellular uptake routes, and clearance mechanisms from the body.^4^, ^5^, ^6^, ^7^, ^8^ For example, while nanoparticles smaller than 100 nm can be taken up via clathrin‐mediated or caveolae‐mediated endocytosis, nanoparticles between 500 nm and 5 μm are taken up via phagocytosis,^5^, ^9^ although often several routes of uptake are possible.^10^ Furthermore, nanoparticles smaller than 8 nm are typically cleared via excretion through the kidneys, nanoparticles between 20 and 150 nm in size are cleared by the liver, and larger than 200 nm nanoparticles are cleared by the spleen.^5^, ^11^ Still, these number should be taken as indicators of orders of magnitude, rather that strict values as other factors also influence clearance. As such, the fabrication of nanoparticles in the nanosize regime and the engineering of their specific sizes and distributions is a significant factor is determining the effectiveness of a carrier system in the body.

A multitude of fabrication methods for the creation of polymeric microparticles and microparticle exists, including various emulsion methods,^12^ particle fabrication in nonwetting templates (PRINT) technology,^13^ self‐assembly of block copolymers,^14^ electrospray techniques,^15^, ^16^ microfluidics,^17^ and layer‐by‐layer assemblies.^18^ Alternatively, electrohydrodynamic (EHD) co‐jetting has been developed as a high throughput system for the fabrication of multifunctional particles and fibers, where the internal architecture can be designed to incorporate unique capabilities.^19^, ^20^, ^21^ During the EHD co‐jetting process, two or more polymeric solutions are flown in a side‐by‐side configuration under a laminar regime, meaning that convective mixing is minimized, and a stable interface between the fluids is achieved in the formed droplet where the two solutions meet. Upon the addition of an electric field, the droplet forms into a Taylor cone, and an electrified polymeric jet is ejected from the very tip that forms into individual droplets. Due to the rapid acceleration of the jet, the immediate reduction in diameter, and the resulting increase in the surface area to volume ratio, the solvents present in the droplets evaporate rapidly, leaving behind solidified particles. As a result of the rapid evaporation of the solvents and the laminar flow regime, the initial flow determined configuration is maintained in the particles in the form of distinct compartments.^21^

In the past decade, the fabrication of multicompartmental fibers and particles with two to seven different compartments and with various shapes via EHD co‐jetting has been well‐established.^22^, ^23^, ^24^, ^25^, ^26^ Additionally, within these systems, the incorporation of water‐based and organic‐based polymers,^25^, ^27^, ^28^, ^29^ functional polymers for the creation of specific targeting and stealth patches on the surface of the particles,^30^, ^31^ stimuli‐responsive polymers for on‐demand therapeutic release kinetics,^32^, ^33^, ^34^ small molecule‐based therapeutics,^33^, ^34^, ^35^ DNA‐based therapeutics,^36^ protein‐based therapeutics,^35^ and imaging agents^22^ have been explored. Furthermore, the interaction of such systems with various cell types,^34^, ^36^, ^37^, ^38^, ^39^, ^40^, ^41^ their biodistribution in vivo,^42^ and their functionality as carriers for dual therapeutic delivery to the cochlea^35^, ^43^ have demonstrated that multifunctional systems fabricated based on EHD co‐jetting can be ideal carriers for targeted delivery in various applications. To date, the majority of these studies have been accomplished using microparticles, and while some have contained nanoparticles,^36^, ^37^, ^42^ a systematic study into the fabrication of uniform, multifunctional nanoparticles using the EHD co‐jetting system has not been reported. In this manuscript, the engineering of nanoparticles with specific size distributions via two different approaches, using charged species or specific solvents, is explored. Upon confirmation of a systematic method for the fabrication of uniformly distributed nanoparticles at specific size ranges, their in vitro uptake as a function of size and incubation period is examined.

## 2 Materials and Methods

### 2.1 Materials for particle fabrication

Chloroform, dimethylformamide (DMF), phosphate buffered saline (PBS), cetyl trimethylammonium bromide (CTAB), poly[tris(2,5‐bis(hexyloxy)−1,4‐henylenevinylene)‐alt‐(1,3‐ phenylenevinylene)] (PTDPV), and tween 20 were used as purchased from Sigma‐Aldrich, USA. Polylactide‐*co*‐glycolide (Purasorb PDLG 5002A) with a ratio of 50:50 lactide to glycolide and a molecular weight of 17 kDa was purchased from Corbion, Inc., USA, and Polylactide‐*co*‐glycolide with a ratio of 50:50 lactide to glycolide and a molecular weight of 17 kDa was purchased from Lakeshore Biomaterials, USA.

### 2.2 Particle fabrication

Particles were fabricated using the EHD jetting procedure. Briefly, the 17 kDa PLGA was dissolved at a 10% wt/vol concentration in various ratios of chloroform and DMF, before being flown in a laminar regime through parallel metallic needles at 0.1 ml per hour. For samples containing CTAB, the charged surfactant was added directly to the polymer solution. Upon the formation of a stable droplet at the interface of the two polymeric solutions, a voltage was applied to the droplet creating a polymeric jet from the tip of the needles. The polymeric jet then split into individual droplets, causing the solvents to evaporate rapidly, leaving behind solidified particles on the grounded electrode. To label the nanoparticles with a fluorescent dye, an organic soluble fluorescent polymer (PTDPV) was dissolved in the particle jetting solution at a 0.1 mg/ml concentration. The particles were then fabricated using the EHD jetting system as described above, as the dye did not require any additional changes to the jetting setup and postprocessing steps. This dye was used due to its strong fluorescent activity (peaking at 518 nm in its emission spectra, similar to fluorescein‐based dye molecules), its hydrophobic nature (chloroform solubility and water insolubility), and its high molecular weight (*M*_w_ = 32 kDa), which together prohibited the release of the dye from the nanoparticles during postprocessing steps and further experiments. Once fabricated, the particles were imaged via Scanning Electron Microscopy (SEM) to determine their shape and size distribution. To quantify the size of the nanoparticles, the ImageJ program was used to measure the size and distribution of over 500 nanoparticles per sample.

### 2.3 Isolation of nanoparticles via centrifugation

Nanoparticles with a solvent ratio of 97:03 chloroform: DMF and 5% wt/vol CTAB were fabricated and collected in PBS and 0.01% tween 20 (vol/vol). The particles were filtered using 40‐μm Falcon cell strainers, sonicated on ice, and centrifuged at 4,000 RPM for 5 min to remove larger impurities. The resulting supernatants were spun at 10,000 RPM for 1, 5, 10, 20, and 30 min time intervals to isolate individual size ranges of nanoparticles. The nanoparticles were characterized by Dynamic Light Scattering (DLS) to determine their size distribution and Nanosight Nanoparticles Tracking Analysis (NTA) to determine their concentration (particles per milliliter).

### 2.4 Materials for cellular uptake studies

Sodium chloride and bovine serum albumin were purchased from Roth and Jackson ImmunoResearch, UK, respectively. Resazurin solution (alamar blue), DyLight 649 donkey anti‐mouse IgG (H+L, secondary antibody), Phalloidin–tetramethylrhodamine B (phalloidin‐TMR), and saponin from quillaja bark (≥10%) were purchased from Sigma‐Aldrich, Germany. The 96‐well assay plates from Corning, Germany, were used for studying the cellular viability. Human cervical carcinoma (immortalized from patient Henrietta Lacks, hence labeled “HeLa”) cells were purchased from American Type Culture Collection, USA, and were seeded in cell culture flasks (25, 75, 150 cm^2^) from Techno Plastic Products, Germany. For cell counting, a Neubauer improved counting chamber (hemocytometer) provided by MARIENFELD Laboratory, Germany, glassware was used. PBS, paraformaldehyde (8%), Hoechst, trihydrochloride, trihydrate, and fluoromount‐G were purchased from Biochrom (Germany), Electron Microscopy Sciences, Life Technologies, and Southern Biotech (Germany), respectively. Lysosomal‐associated membrane protein 1 (LAMP 1; mouse anti‐human IgG1; developmental studies hybridoma bank, Supernatant) was purchased from the University of Iowa, Department of Biology. Glycine (≥99%), sterilized 10‐mm round cover slips with thickness of 0.17 ± 0.005 mm, glass slides (76 × 26 mm), and parafilm were purchased from Carl Roth, Germany. The 4‐well tissue culture plates were obtained from Thermo Scientific, Germany.

### 2.5 Nanoparticle stability studies

The colloidal stability of nanoparticles of different average diameters (*d*_1_ = 174 nm, *d*_2_ = 378 nm, and *d*_3_ = 575 nm, as determined by DLS) was studied^44^ in the presence of different concentrations of sodium chloride and bovine serum albumin. The variation in the hydrodynamic diameters (*d*_h_) of nanoparticles was used as an indicator of their stability in saline and protein rich environments, respectively.^45^ Nanoparticles were exposed to varying concentrations of sodium chloride in water (0–5 M) and bovine serum albumin in PBS (0–800 µM). Final concentrations of nanoparticles after mixing with different concentrations of NaCl and BSA were 2 × 10^9^ and 4 × 10^9^ nanoparticles/ml, respectively. The hydrodynamic diameters of the nanoparticles were measured three times via DLS analysis and are presented with corresponding standard deviations in Supporting Information Figure 1.

### 2.6 Cellular toxicity studies

For determining the acute impact of the exposure of nanoparticles on cellular viability, a resazurin‐based cytotoxicity assay was performed, which is based on the mitochondrial activity of the living cells.^46^, ^47^ Active mitochondria of the living cells perform the bioreduction of the dye, that is, they convert the nonfluorescent blue dye (resazurin) into its reduced form (resorufin) which fluoresces pink. These studies were performed by seeding HeLa cells in 96‐well transparent bottom plates (7,500 cells/well, area of each well was 0.32 cm^2^) in 100 µl of complete growth media (Dulbecco's Modified Eagle Medium (DMEM) supplemental with 10% FBS, 1% P/S, and 1% glutaMAX^TM^) and incubated for 24 hr at 37°C with a constant supply of 5% CO_2_. After 24 hr, when HeLa cells had adhered to the bottom of 96‐well assay plates, the old growth media was replaced by fresh growth media containing nanoparticles at different concentrations c(NP). Serial dilutions of nanoparticles were performed to examine the toxic effect for a range of nanoparticle concentrations (1 × 10^10^−1 × 10^7^ nanoparticles/ml) and each dose was added in triplicate. In a few wells of the assay plates, fresh growth media (without nanoparticles) was added to the cells, which served as positive control. After 24 hr of incubation of the cells with the nanoparticles, the growth media was aspirated and the cells were washed with PBS followed by the addition of 100 µl of 10% resazurin solution in complete cell growth media into each well of the assay plates. Resazurin solution (10%) was added in three wells of the assay plates (without cells), which served as negative control and the assay plates were incubated for 3.5 hr under the aforementioned conditions. After 3.5 hr of incubation, the fluorescence spectra of each well of the assay plates were recorded via spectrofluorometer coupled with a microwell‐plate reader using an excitation wavelength of 560 nm and acquiring the emission spectra from 572 to 650 nm. The mean of the maximum fluorescence intensity values for each concentration was determined and the viability for each nanoparticle concentration‐treated cell was defined as the mean of the maximum fluorescence intensity of resorufin (originating from each well of the assay plate). For background correction, the mean of background values was subtracted from the mean of maximum fluorescence intensity values for each concentration. Finally, all values obtained were normalized with respect to their positive controls (cells without nanoparticles).

### 2.7 Cellular uptake of nanoparticles

HeLa cells were initially grown in 75 cm^3^ flasks and were seeded for nanoparticle uptake on sterilized round glass cover slips (diameter = 10 mm) placed into 4‐well cell culture plates at a density of 20,000 cells per well. Each well had a surface area of 1.9 cm^2^ and was filled with 0.5 ml of complete cell growth medium (DMEM supplemented with 10% FBS, 1% Glutamax, and 1% P/S). Cells were grown at 37°C in an incubator with a constant supply of 5% CO_2_. After 24 hr, the growth media was replaced by fresh growth media containing nanoparticles at a final concentration of 1.5 × 10^8^ nanoparticles/ml. Nanoparticles were shortly sonicated for 30 s just before mixing with cell growth medium and cells were exposed to nanoparticles for different time intervals (6, 12, and 24 hr) under the aforementioned conditions. In parallel, control experiments were also performed in which cells were grown in the presence of complete cell growth media without addition of nanoparticles.

After incubation with nanoparticles for defined time intervals, the cells were washed with PBS and fixed with 4% paraformaldehyde solution in PBS (20 min incubation at room temperature), followed by three washes with PBS. The fixed cells were transferred on Parafilm previously spread on an unmodified surface and were exposed to permeabilization solution (glycin 5 mg/ml and saponin 0.5 mg/ml, in PBS) for 5 min at room temperature, followed by treatment with blocking solution (20 mg/ml BSA in the permeabilization solution) for 30 min at 37°C. Cells with nanoparticles were immunostained by means of LAMP 1 (2 µg/ml, primary antibody in blocking solution), incubated for 1 hr at 37°C, washed three times with blocking solution and exposed to a solution of Dylight 649 conjugated donkey anti‐mouse (1.25 µg/ml, secondary antibody), Hoechst 33342 (0.5 µg/ml), and phalloidin‐TMR (40 nM) in PBS for another 1 hr at 37°C. Hoechst 33342 and phalloidin‐TMR were used to stain cellular nuclei and cytoskeletons, respectively.^10^, ^48^ Afterward, the cells were washed three times with PBS, one time with water and after mounting with Fluoromont‐G on glass slides (76 × 26 mm) were placed in a dark and dry place for 24 hr.

A Confocal Laser Scanning Microscope (CLSM 510 Meta) from Zeiss was used for visualizing the fixed and immunostained cellular samples containing internalized nanoparticles. For sample visualization and image acquisition, the CLSM was equipped with diode, argon, and helium neon lasers and excitations of 405, 488, 543, and 633 nm were used for all imaging. Fluorescence micrographs of immunostained samples with internalized nanoparticles and their corresponding controls (cells without nanoparticles) were captured. The nuclei stained with Hoechst reagent were excited at 405 nm and the dye emission was detected between 420 and 480 nm. Nanoparticles containing the PTDVP dye were visualized by exciting at 488 nm and detecting the emission between 505 and 550 nm. The fluorescence of the phalloidin‐TMR labeled cytoskeleton was excited at 543 nm, and emission was captured using a 560‐nm long pass filter. Antibody‐labeled lysosomes were excited at 633 nm, and their emission was recorded via a 650‐nm long pass filter.

In detail, z‐stacks/three‐dimensional (3D) image stacks (with 0.1 µm resolution in the *xy*‐plane and 0.48 µm resolution along the *z* axis) were acquired using a pinhole aperture of 1 Airy unit. Thirty to fifty images were recorded per sample covering an average of 60–100 cells per condition. Images of control samples were acquired to set threshold values for image processing and data evaluation of nanoparticle‐treated samples. To assess the number and intracellular location of internalized nanoparticles from fluorescence image stacks a similar procedure was applied as proposed by Torrano et al.^49^, ^50^

The uptake of nanoparticles by cells, in particular their intralysosomal fraction, was quantified by Digital Image Cytometry^51^, ^52^ using the fluorescence micrographs captured by CLSM.^48^ Each cell (including the nucleus, lysosomes, and associated nanoparticles) was reconstructed and modeled in 3D using MATLAB (Mathworks) and CellProfiler^53^ by applying the following approach: based on the two‐dimensional (2D) plane (around *z* = 0 µm) where cells showed the largest cross‐sectional area, the outlines of the individual cells were identified as described by Pelaz et al.^54^ These outlines were expanded along the *z*‐dimension to separate touching cells reliably by die‐cutting the reconstructed volumetric data at a later stage. Prior to modeling, the images in different fluorescence channels of the stack underwent noise reduction by 3D‐median filtering with a kernel size of 3 × 3 × 3 voxels. In case of nuclei and cytoskeletal signals, the images were slightly smoothed after noise reduction with a 3D Gaussian filter of size 5 × 5 × 3 pixels. Then, all slices were binarized by manual thresholding. Following this, morphological operations were applied to improve the quality of the 3D reconstruction (cleaning the image stack from very small clusters consisting of less than 4 × 4 × 4 connected voxels and filling holes in the remaining structures). In case of the cytoskeletal stain, the images were morphologically closed with a three‐voxel‐sized diamond‐shaped structuring element after binarization to avoid ruptures in the reconstructed cell surface. The images of the channel containing the nanoparticle signals were treated exactly as described earlier. Finally, all connected voxels were identified yielding a volumetric representation of nuclei, cells, lysosomes, and aggregates of nanoparticles.

For each aggregate of nanoparticles (in terms of connected voxels), the total integrated fluorescence intensity was determined (to calculate the amount of nanoparticles per aggregate) and the relative position with respect to the cell and lysosomal structures was calculated. Nanoparticle aggregates located in the extracellular areas were not considered (ie, center of mass of aggregate was outside the cell body volume). Nanoparticles overlapping with lysosomal structures (ie, the center of mass of the aggregate of nanoparticles was inside the lysosomal volume) were classified as being located inside the lysosomes. Based on these assumptions the number of nanoparticles per cell, *N*_cell_, and the fraction localized inside the lysosomes, *N*_lyso_, was calculated. The internalization results are expressed as median values ± upper/lower quartile. However, larger particles (clusters/clumps of nanoparticles) with high fluorescence intensity were also detected while quantifying the internalization of nanoparticles, the presence of which might hamper the precision of the evaluated data.

## 3 Results and Discussions

**Figure 1:**
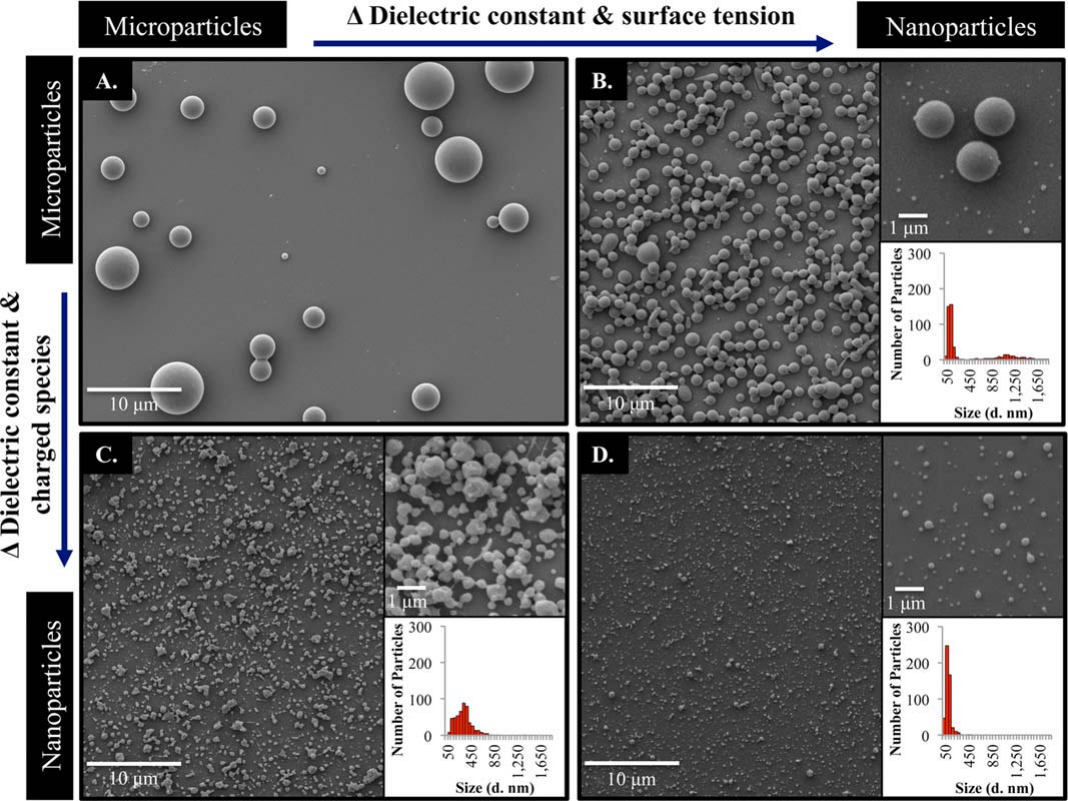
Using the same polymer and concentration, particles ranging in diameter (*d*) from micrometers (A) to nanometers (D) were prepared. By increasing the amount of DMF, polydisperse microparticles (A) could be downsized to bimodal particles where one set was at approximately 1 μm and one at 100 nm (B). Alternatively, by adding a charged surfactant, polydispersed microparticles (A) could be downsized to polydispersed nanoparticles ranging in size from 50 to 800 nm (C). Combining these effects resulted in monodispersed nanoparticles in the 50–150 nm range (D). The insets in each image represent a higher magnification image of the particles (top) and the size distribution of the particles based on ImageJ analysis of SEM images (bottom)

**Figure 2:**
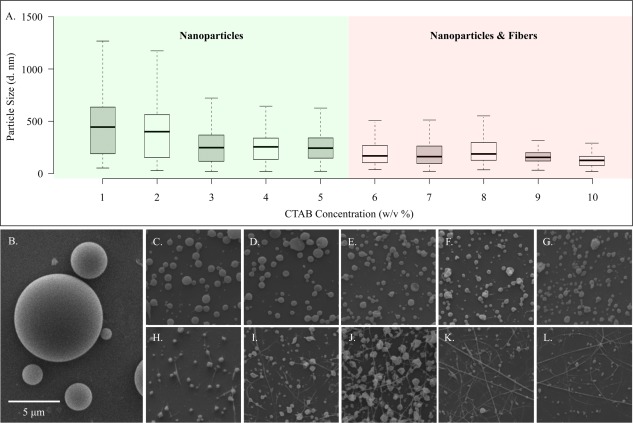
Effect of CTAB concentration on the diameter (*d*) and distribution of nanoparticles when the polymer concentration and solvent ratios are kept constant. (A) Box plot of nanoparticle size distributions, and (B–L) are representative SEM images of particles with 0–10% wt/vol of CTAB, respectively

**Figure 3:**
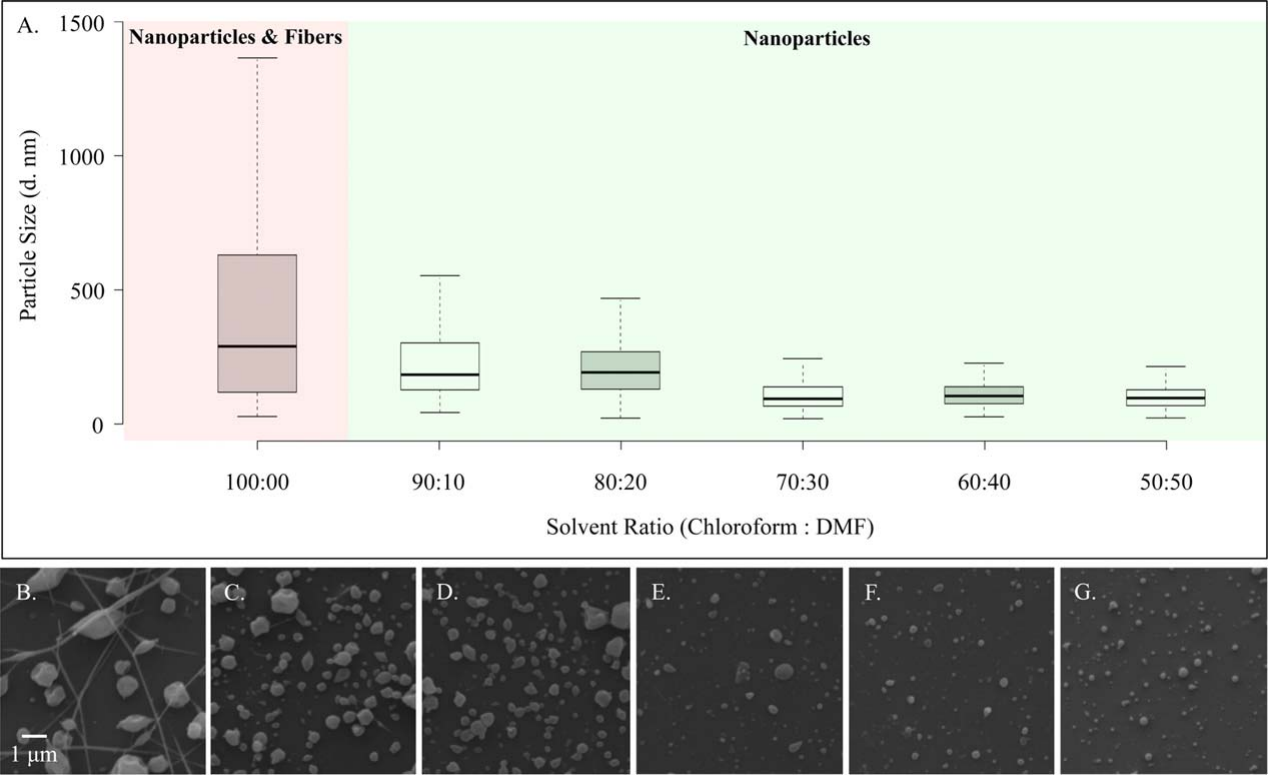
Effect of solvent ratio on the size (in terms of particle diameter *d*) and distribution of nanoparticles when the concentration of the polymer and charged species are kept constant. (A) Box plot of nanoparticle size distributions and (B–G) are SEM images of nanoparticles with a decreasing amount of chloroform to DMF ratio of 100:00 to 50:50, respectively

Size is an important physical parameter for particles used in drug delivery applications due to its influence over circulation times, rates of opsonization, cellular uptake, passive targeting to tumors via the EPR effect, tumor penetration, and excretion from the body.^55^, ^56^ In the EHD co‐jetting system, several parameters such as polymer molecular weight and concentration, solvent viscosity and dielectric constant, and the applied voltage mainly control the size and uniformity of particles ranging from micrometer to nanometer in diameter. One of the most common routes of fabricating nanoparticles and nanofibers via electrospraying, is the use of polymeric solutions with dilute concentrations.^57^, ^58^, ^59^, ^60^ Based on this, our initial studies on the fabrication of nanoparticles focused on the use of polymers with low molecular weight values (4.1 kDa) and at dilute concentrations (0.01–1% wt/vol). While these experiments did result in nanoparticles as small as 100–200 nm (Supporting Information Figure 1), the yield was extremely low for this process due to the dilute concentrations used (as a reference, the images shown in Supporting Information Figure 1 were taken after 4–10 hr of jetting on the same substrate (S. F. 1 A‐C, respectively), where for all images shown in Figures 1, 2, 3, the jetting was only done for 30 min). As a result, other routes of fabricating nanoparticles were explored to establish a fabrication procedure that could result in higher yields.

The other common parameters investigated for the fabrication of nanoparticles include the use of solvents with higher dielectric constants/surface tensions and the incorporation of charged species, which affect the overall charge of the jetting solutions. It has been well established that the use of solvents with high dielectric constants, such as *N*, *N*‐dimethyl formamide (DMF), can result in the fabrication of nanofibers rather than microfibers due to the induced higher net charge density on the jetting solution.^61^, ^62^, ^63^ Similarly, the use of a charged surfactant results in an overall increase in the charge density and conductivity of the jetting solution, which ultimately results in nanofibers and particles with smaller diameters.^15^, ^62^, ^64^, ^65^ Building on these studies, the effect of each parameter, using the same poly (lactide‐*co*‐glycolide) polymer (PLGA 50:50, 17 kDa) and at the same concentration of 10% wt/vol, on particle size and their combined effects were subsequently studied. It was observed that a solvent ratio of 97:03, chloroform:DMF yielded polydispersed microparticles (Figure 1A), while the same solution with a solvent ratio of 1:1, chloroform:DMF yielded bimodal, yet uniform particles with one set of nanoparticles at approximately 50–150 nm and a second set at approximately 1 μm (Figure 1B). Alternatively, keeping the same ratio of solvents (97:03), but adding a charged surfactant, CTAB, at 5% wt/vol resulted in a polydispersed population of nanoparticles ranging from 50 to 800 nm (Figure 1C). Combining these two parameters (5% wt/vol CTAB addition and a ratio of 1:1 chloroform: DMF) resulted in monodispersed nanoparticles ranging from 50 to 150 nm in diameter (Figure 1D). Higher magnification images and the size distribution of the nanoparticles based on ImageJ analysis of the SEM images are included as insets for each set of particles.

To fully understand the effect of each of these parameters (charged species and solvent with higher dielectric constant), a more systematic study was conducted, where each parameter was tested at an increasing concentration or ratio, while keeping all other parameters constant. To study the effect of the charged species, the concentration of the CTAB was increased from 0 to 10% wt/vol at 1% intervals, while the polymer concentration, molecular weight, and solvent ratio (97:03 Chloroform:DMF) were kept constant in order to isolate the effect of the charged species.

The resulting particles were imaged with SEM and their size distribution was determined based on analysis with the program ImageJ. As demonstrated in Figure 2, the median values and the size distributions for the nanoparticles decrease as the CTAB concentration is increased (the median value and size distribution of the zero percent particles are not included in the graph due to the much larger values of 3,255 nm and 450–16,710 nm, respectively). However, there is a limit to the amount of CTAB that can be used in a given solution: at higher values than 5% wt/vol, the jetting solution results in the fabrication of a mixture of nanoparticles and fibers, instead of the desired smaller sized nanoparticles. Additionally, the stability of the jetting solution and the yield of the nanoparticles also decreases with increasing CTAB concentrations. As a result, the optimum formulation for fabricating nanoparticles by solely relying on the effect of a charged species is approximately 4–5% wt/vol. In addition, CTAB can be cytotoxic and thus its use at high concentrations is not desirable.

A similar study was conducted to determine the optimum ratio of chloroform to DMF for the engineering of uniform nanoparticles at specific size ranges. Here, the polymer concentration, molecular weight, and CTAB concentration (5% wt/vol) were kept constant, while the ratio of chloroform and DMF was decreased from 100:00 to 50:50, respectively (Figure 3). Similar to nanoparticle formulations with higher than 5% wt/vol of CTAB, the formulation with chloroform as the sole solvent resulted in a sample with fibers and particles. For formulations with DMF, nanoparticles without any fibers could be fabricated, which narrowed in size as the ratio of chloroform to DMF was decreased. As demonstrated in Figure 3, a distinct difference in size distribution can be seen after the 80:20 chloroform to DMF ratio, where both the median size and the distribution drops significantly. As a note, while not much difference is observed in the median and distribution of the samples with 30, 40, and 50% DMF ratios, the yield of the nanoparticles did decrease with increasing DMF concentration. This is most likely due to the insolubility of CTAB in DMF and the instability in the Taylor cone associated with this. Thus, the samples with the higher yields and lowest nanoparticle size and distribution were achieved with the 70:30 and 60:40 chloroform to DMF ratios. By employing DMF, a much more uniform population of nanoparticles as compared to the CTAB samples could be engineered. As such, it would appear that while CTAB can be used to reduce the size of the particles from micron‐sized to nanosized, the incorporation of higher DMF ratios can be used to engineer nanoparticles with more uniform size distributions.

**Figure 4:**
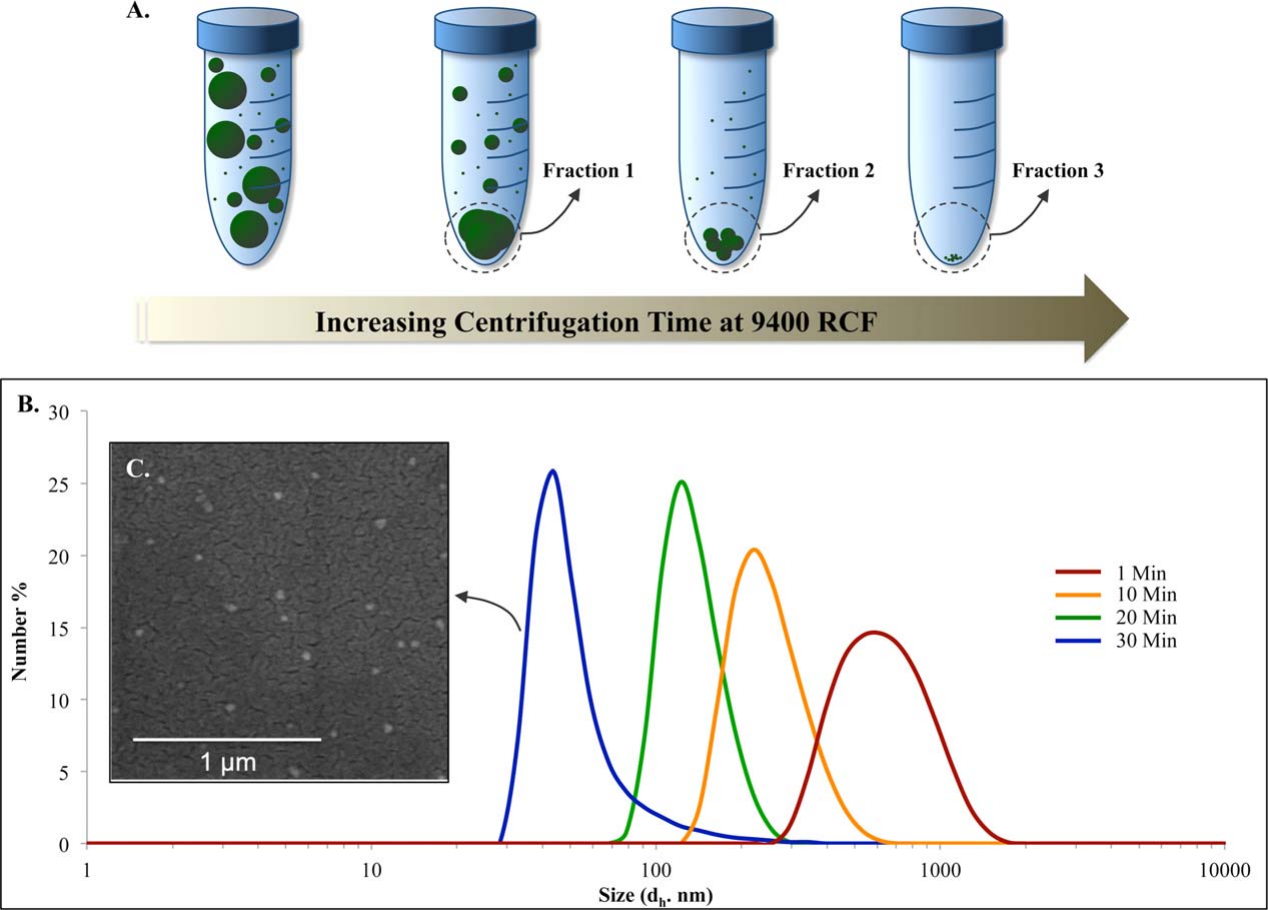
Separation of a polydispersed group of nanoparticles into monodispersed fractions using serial centrifugation. (A) Schematic displaying serial centrifugation. (B) Size distribution (in terms of hydrdodynamic particle diameter (*d*_h_) of different fractions (1, 10, 20, and 30 min) based on DLS measurements. (C) Representative image of uniform 50‐nm nanoparticles from the 30‐min sample

In addition to fabricating monodispersed nanoparticles, the isolation of nanoparticles with specific size ranges from a polydispersed sample can be of interest, especially as it allows for the side‐by‐side testing of nanoparticles with the same properties (same batch, material, and physical characteristics), but with different size ranges. This fractionation can be achieved by using serial centrifugation (Figure 4A), where a polydispersed sample of particles (here the sample in Figure 1C) is centrifuged at a set force of 9,400 relative centrifugal force for longer durations to pellet increasingly smaller nanoparticles. The size distribution of several fractions (1, 10, 20, and 30 min) with median sizes of 44, 122, 220, and 615 nm are shown in Figure 4B, as analyzed by DLS. In addition, a SEM image of the 30 min sample showing nanoparticles with a size distribution of 30–110 nm is demonstrated as an inset in Figure 4C. Here, the hydrodynamic diameter (*d*_h_) as measured by DLS is displayed as oppose to the diameters shown in previous figures that were measured by ImageJ analysis of SEM images (denoted as “*d*” in this manuscript).

As mentioned previously, the isolation of particles with specific size ranges from the same batch of particles, rather than their individual fabrication by changing the jetting parameters, allows for the side‐by‐side study of their behavior solely as a function of size. Note that such fractionation techniques can be very precise, allowing for the isolation of discrete particle species.^66^ We were specifically interested in employing this technique to study the effect of size on the cellular uptake of nanoparticles. To begin, nanoparticles with average hydrodynamic diameters of 174, 378, and 575 nm were isolated via serial centrifugation and their colloidal stability in buffered solutions containing various salt (sodium chloride at 0–5 M) and protein (bovine serum albumin at 0–800 µM) concentrations were studied (Supporting Information Figure 2).^44^ The variation in the hydrodynamic diameter (*d*_h_) of the nanoparticles was analyzed by DLS measurements and used as an indicator of their stability. While the nanoparticles were stable in sodium chloride concentrations of less than 0.34 M, at higher concentrations the size of the nanoparticles increased as they started to aggregate in the solution. Such behavior is well known for most colloids.^67^ However, as typical physiological concentrations of sodium chloride in the body are at 0.15 M, all three nanoparticle samples are expected to be colloidally stable for drug delivery applications as with regard to the salt concentrations.

Incubation studies with albumin were conducted to study the effect of the creation of a protein corona on the stability of these nanoparticles in solution.^45^ The nanoparticles, especially the two larger sets of nanoparticles, were shown to be relatively stable in increasing concentrations of the albumin and increased in size as the concentration of albumin, and the size of the protein corona, increased. The effect of the protein corona was more pronounced on the smaller nanoparticles as the protein corona would be more obvious on the smaller sized particles (ie, a protein corona of 50 nm is more pronounced on 100 nm particles [50% size increase] than on 500 nm particles [10% size increase]). While the nanoparticles are prone to building a protein corona, this can potentially be mitigated by incorporating stealth moieties as surface modifications in future studies.

**Figure 5:**
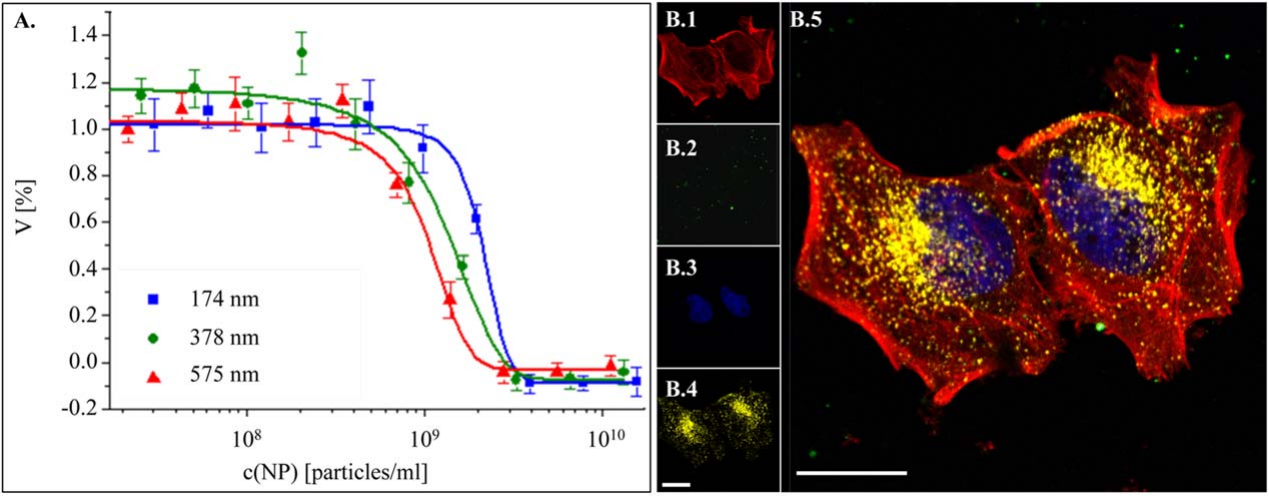
Cellular uptake of nanoparticles. (A) Cell viability (*V*) of HeLa cells upon 24 hr of exposure to nanoparticles as a function of nanoparticle concentration c(NP). Data present the mean values of three experiments with corresponding standard deviations. (B) A representative CLSM image of nanoparticles internalized by HeLa cells after 12 hr of incubation. Stained cellular compartments are shown as phalloidin‐TMR conjugated cytoskeleton (B.1, red), fluorescently labeled nanoparticles (B.2, green), Hoechst stained cellular nuclei (B.3, blue), immunostained lysosome (B.4, yellow), and an overlay of all channels showing aforementioned cellular compartments and nanoparticles (B.5). The scale bars correspond to 20 µm

Before conducting in vitro studies to determine the effect of size on nanoparticle uptake, the cytotoxicity of the nanoparticles was determined. A resazurin‐based assay was used to demonstrate the cytotoxicity by incubating known concentrations of nanoparticles (particles per milliliter as determined by Nanosight's NTA technique) with HeLa cells. The resazurin assay is based on the mitochondrial activity of living cells, where the active mitochondria perform the reduction of the dye that can be detected via a plate reader.^46^, ^47^ In other words, the fluorescence intensity of resorufin can be directly related to the viability of the cells. Note that this test has to be regarded as a basic screening for the acute toxic effects of nanoparticles on cells, whereas for more profound analysis more sophisticated tests are necessary. In particular, due to the short incubation times only severe acute reduction in cellular viability can be probed. The viability studies were performed by seeding HeLa cells for 24 hr, followed by incubation with nanoparticles at various concentrations (10^7^ to 10^10^ nanoparticles per ml). After a 24‐hr incubation, the resazurin assay determined the number of live cells as compared to controls that were not incubated with nanoparticles. From these studies (Figure 5A), it was determined that none of the nanoparticle fractions imposed acute cytotoxicity to HeLa cells at concentrations below 4·× 10^8^ nanoparticles per ml. As such, nanoparticle uptake studies with the HeLa cells were done at a concentration of 1.5 × 10^8^ nanoparticles per ml, in order to ensure that toxicity would not be a factor in the studies.

To study the cellular uptake of nanoparticles as a function of size, the three separate nanoparticle populations (average hydrodynamic diameters of 174, 378, and 575 nm) were incubated with HeLa cells for 6, 12, and 24 hr. After the incubations, the cells were fixed, and their cytoskeleton, nuclei, and lysosomes were immunostained.^10^ The samples were analyzed with confocal microscopy to view the uptake of the nanoparticles as a function of size over time. An example of the 2D images obtained from these studies is demonstrated in Figure 5B.1–5, where the cytoskeleton (red), nanoparticles (green), nuclei (blue), lysosomes (yellow), and their overlay image can be seen, respectively. Here, the 378 nm nanoparticles were incubated for 12 hr with HeLa cells. Corresponding control studies, where nanoparticles were not incubated with the cells, were also done for later correlation studies to set the threshold values for the image processing and data evaluation of the nanoparticle treated cells (an example of these studies at the 12‐hr incubation without nanoparticles is displayed in Supporting Information Figure 3).^48^

**Figure 6:**
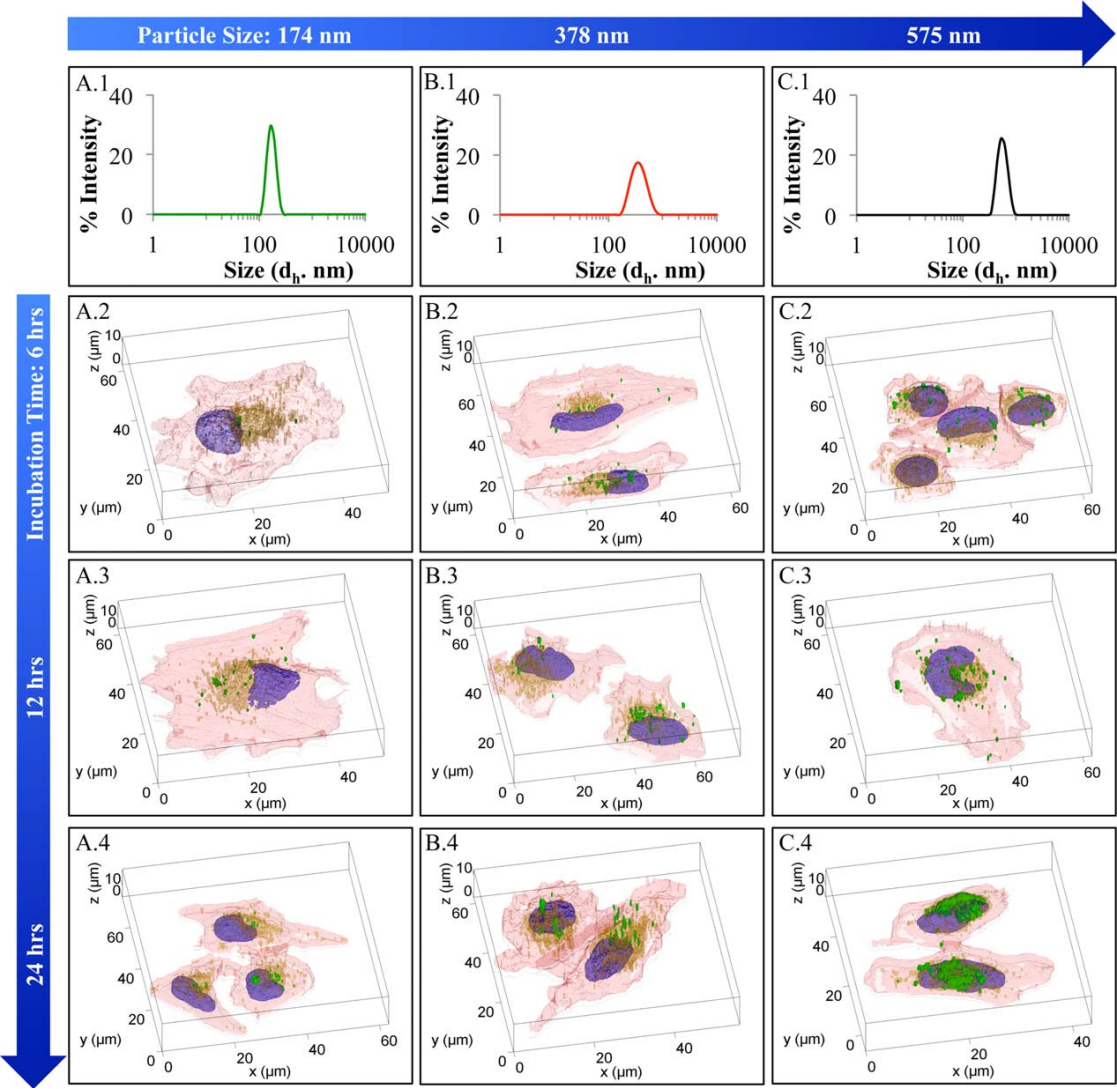
Cellular uptake of nanoparticles with different hydrodynamic diameter (*d*_h_) as a function of time. (A.1–C.1) Size distribution of the nanoparticles and (A.2–C.4) nanoparticle uptake by cells after 6, 12, and 24 hr as demonstrated by 3D reconstructions of confocal images, respectively. Here, the cytoskeleton (red), nuclei (blue), lysosomes (yellow), and nanoparticles (green) are demonstrated

Additionally, images at different focal planes were also obtained for each of the samples, which were then reconstructed into 3D images as demonstrated in Figure 6. Here, the DLS data in Figures 6A.1, 6B.1, and 6C.1 display the size distribution of each set of nanoparticles, followed by the 3D reconstructed images of the cellular uptake of the nanoparticles as a function of time (6, 12, and 24 hr). Similar to the 2D images in Figure 5, here the nucleus (blue), cytoskeleton (red), lysosomes (yellow), and nanoparticles (green) are demonstrated. As can be observed for all three size fractions, the number of nanoparticles associated with cells increase as the duration of nanoparticle incubation increases. Longer incubation time allows for a larger portion of the cells to come into contact with the nanoparticles and permits multiple routes of nanoparticle uptake to take place. This is especially important for the larger sized nanoparticles, as their mode of uptake may be different due to their larger size.^5^, ^9^

To quantify this data and determine the absolute number of internalized fluorescent nanoparticles per cell, an average integrated florescence signal per nanoparticle was determined for each nanoparticle type.^48^ For this purpose, a z‐stack of a drop of diluted nanoparticle suspension was acquired and embedded in fluoromount‐G to mimic the conditions used for cell imaging, and to inhibit Brownian motion. For determining the distribution of integrated fluorescence intensity per nanoparticle, individual nanoparticles were segmented applying the following procedure: (a) the photon shot noise was removed by 3D‐median filtering with a kernel size of 3 pixels, (b) the image was converted by manually defining the threshold, and (c) the morphological operations were applied to improve the quality of the 3D reconstruction. Finally, the connected components in the resulting matrix were identified and the underlying fluorescence signal of each voxel cluster (referring to one or more nanoparticles) was summed to determine the distribution of the integrated intensity values per nanoparticle.^48^ The maxima of the resulting distribution functions were picked as average integrated intensity per nanoparticle (Supporting Information Figure 4).

**Figure 7:**
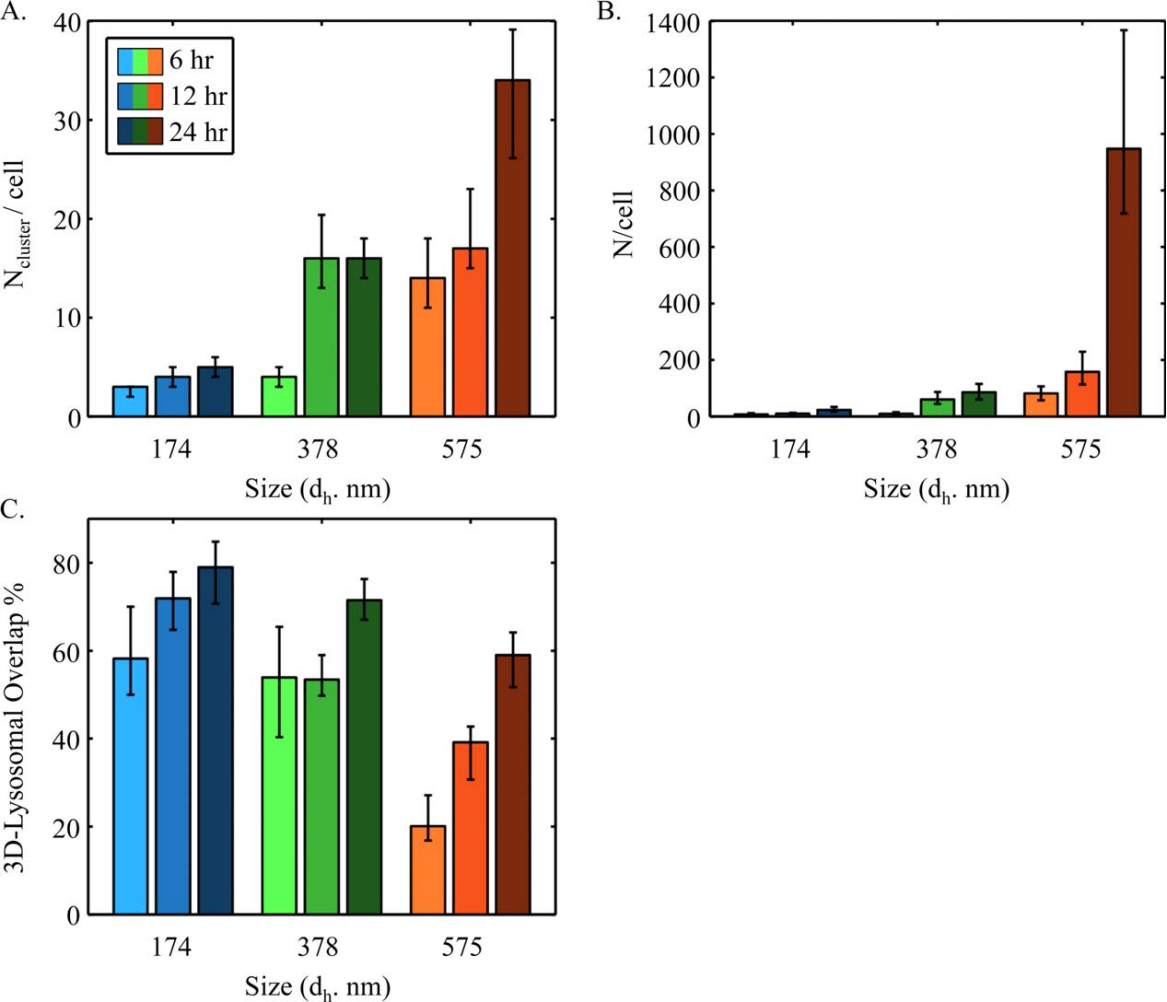
Quantification of nanoparticle uptake by cells. The data are displayed for the number of nanoparticle clusters per cells (A), number of nanoparticles per cells (B), and 3D lysosomal overlap percentage (C)

Based on this information, and the obtained volumetric cell data from confocal imaging, the total number of internalized nanoparticles per cell could be calculated (Figure 7). Here, the volumetric cell data are the number of nanoparticle clusters, which is proportional to the volume of incorporated nanoparticles, and the total number of internalized nanoparticles is the volume of internalized nanoparticles divided by the volume of one nanoparticle. As explained in the methods section, it was automatically distinguished whether a nanoparticle was adhering to the cellular plasma membrane or already fully internalized. Additionally, the nanoparticles were correlated spatially with lysosomes for further analysis. In general, greater enrichment of nanoparticles inside the cells and lysosomes was observed with the passage of time as the nanoparticles have more opportunity to come into contact with the cells and be taken up (Figure 7A). Additionally, greater intralysosomal localization of the nanoparticles with the smallest sized nanoparticle set (average of 174 nm) was observed, followed by sets in increasing sizes (averages of 378 and 575 nm), most likely due to the faster endosomal uptake of smaller nanoparticles (Figure 7C). In addition, the internalization of nanoparticle clusters and number of nanoparticles per cell were the highest for the largest nanoparticle size. We note that for such studies the metrics of uptake is paramount, which in this case was chosen as the number of internalized nanoparticles per cell.^68^ Nevertheless, the outcome is somewhat unexpected and future studies will need to focus on addressing the underlying mechanism.

## 4 Conclusions

The fabrication of nanoparticles and the engineering of their size and distribution is an important factor in the design and success of carriers for drug delivery applications. Here, the fabrication of nanoparticles via the EHD co‐jetting technique using three different strategies (low concentrations, solvent choice, and charged species) and their resulting nanoparticles is explored. It is demonstrated that while using low concentrations of polymeric solutions can result in nanoparticles, the yields are low. Alternatively, uniform nanoparticles with high yields can be fabricated by the incorporation of a charged species, CTAB, and a solvent with a high dielectric constant, DMF, which result in size reduction and uniformity, respectively. Furthermore, a polydispersed population of nanoparticles was fractionated into specific size ranges via serial centrifugation to study their cellular uptake as a function of their size, while keeping all other parameters constant. The uptake of the nanoparticles over time (6, 12, and 24 hr) and their association with lysosomes were investigated. Based on these studies, it is demonstrated that uniform nanoparticles in high yields can be prepared based on the EHD co‐jetting technique and that such nanoparticles can be of value for in vitro and in vivo studies for specific applications.

## Supporting information

Additional Supporting Information can be found the online version of this article at the publisher's website.

Supporting Information

Supporting Information

Supporting Information

Supporting Information

Supporting Information
