# Supplementary figures and images for: Engineering of nanoparticle size via electrohydrodynamic jetting
Source: Bioeng Transl Med. 2016 Jun 20;1(1):82–93. doi: 10.1002/btm2.10010 (PMC5689507; doi:10.1002/btm2.10010)

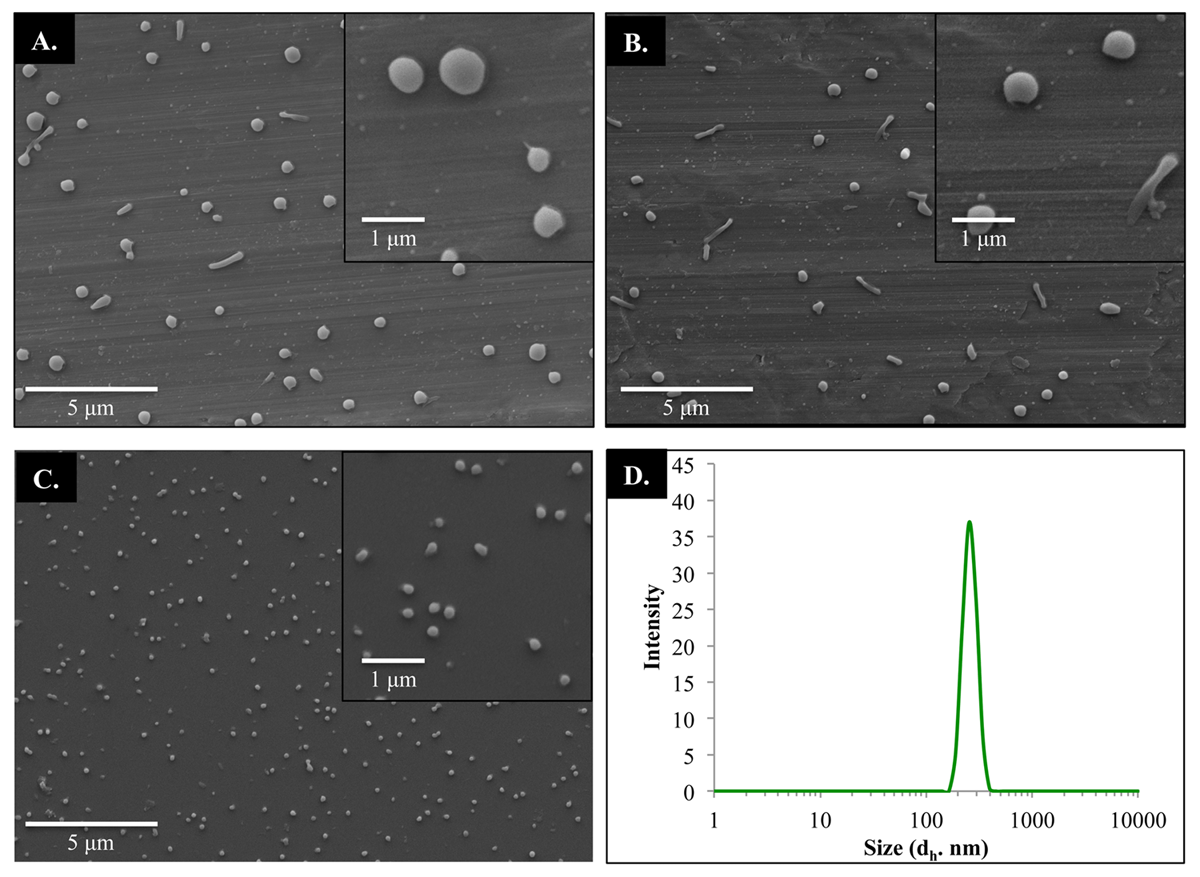

Supplement: Supplementary file 1 — Supporting Information [file BTM2-1-082-s001.tif]

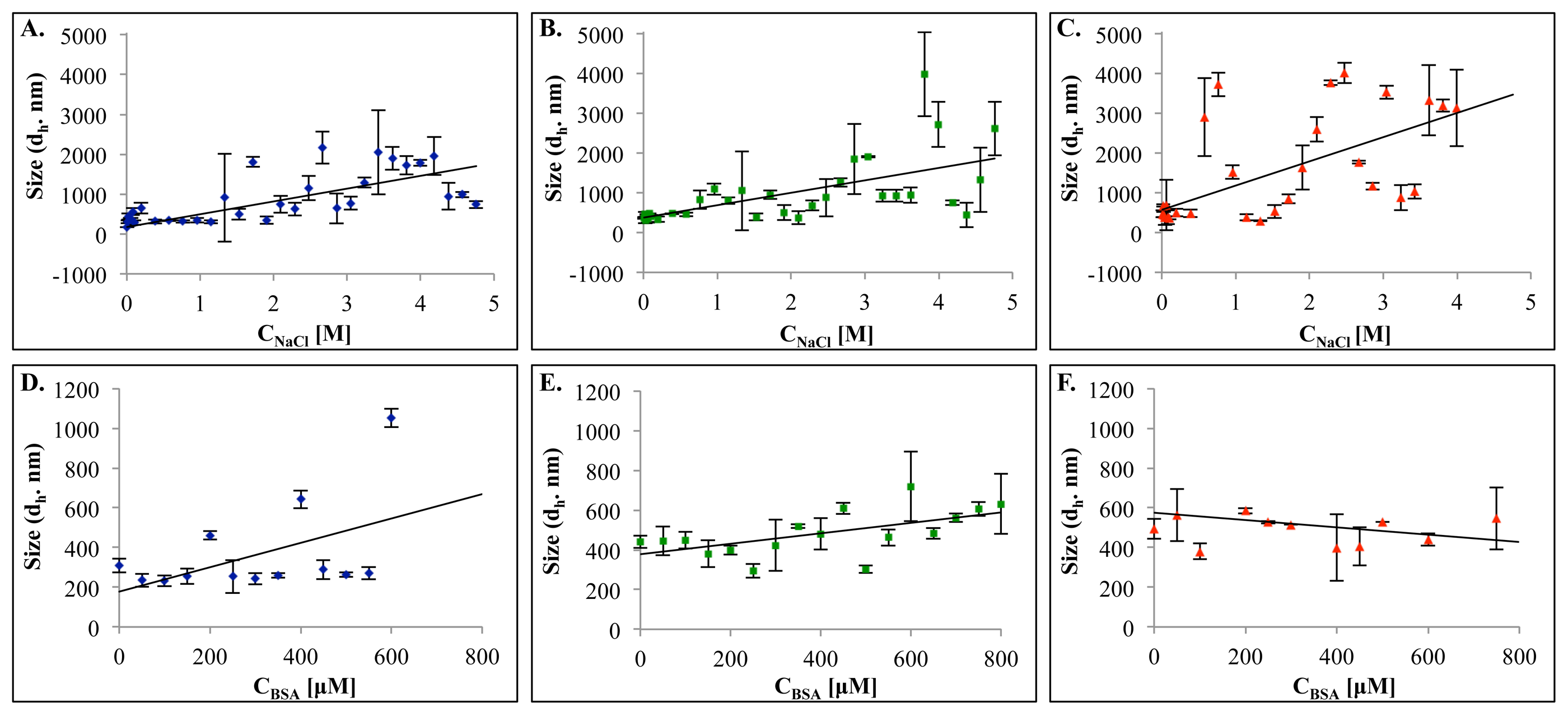

Supplement: Supplementary file 2 — Supporting Information [file BTM2-1-082-s002.tif]

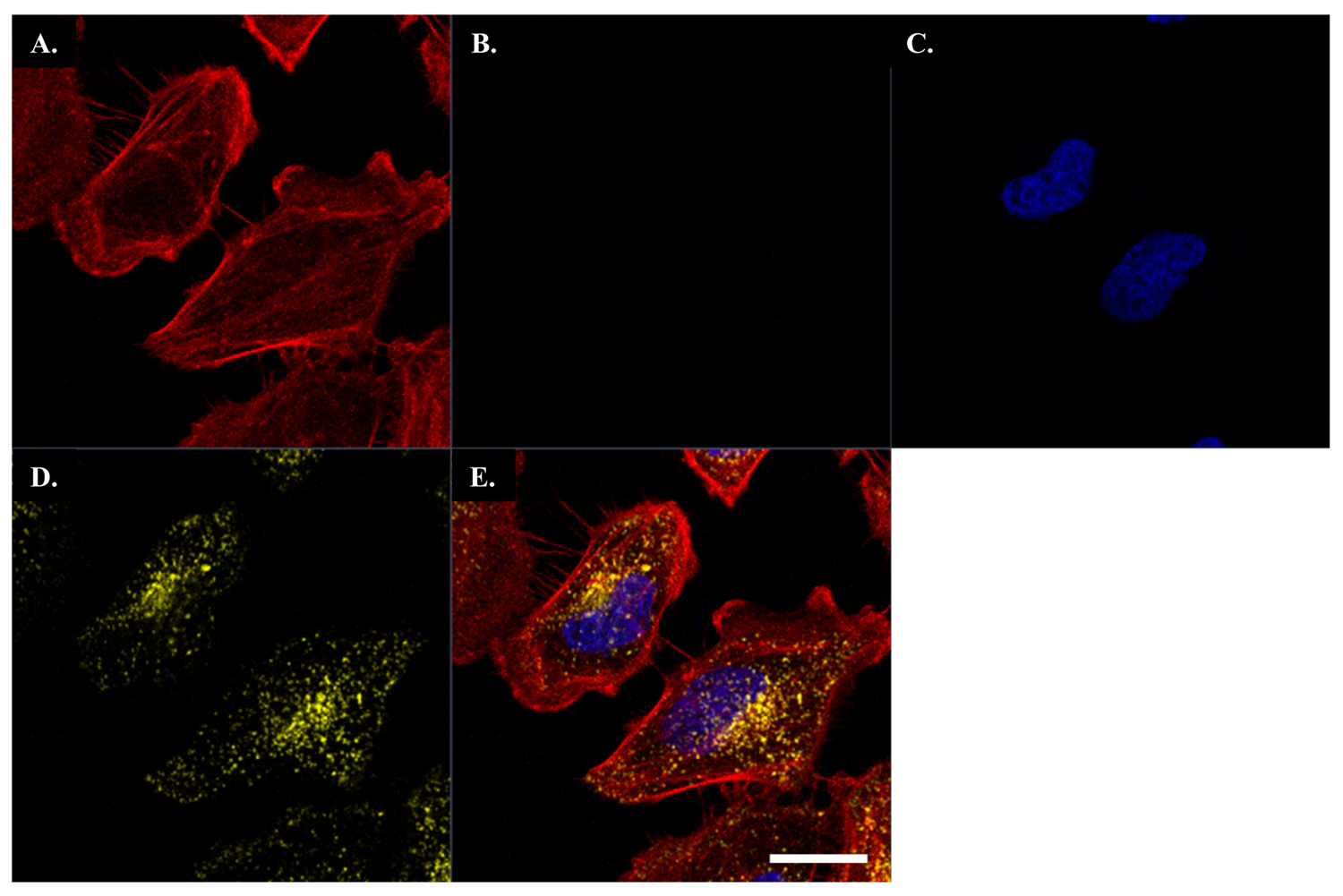

Supplement: Supplementary file 3 — Supporting Information [file BTM2-1-082-s003.tif]

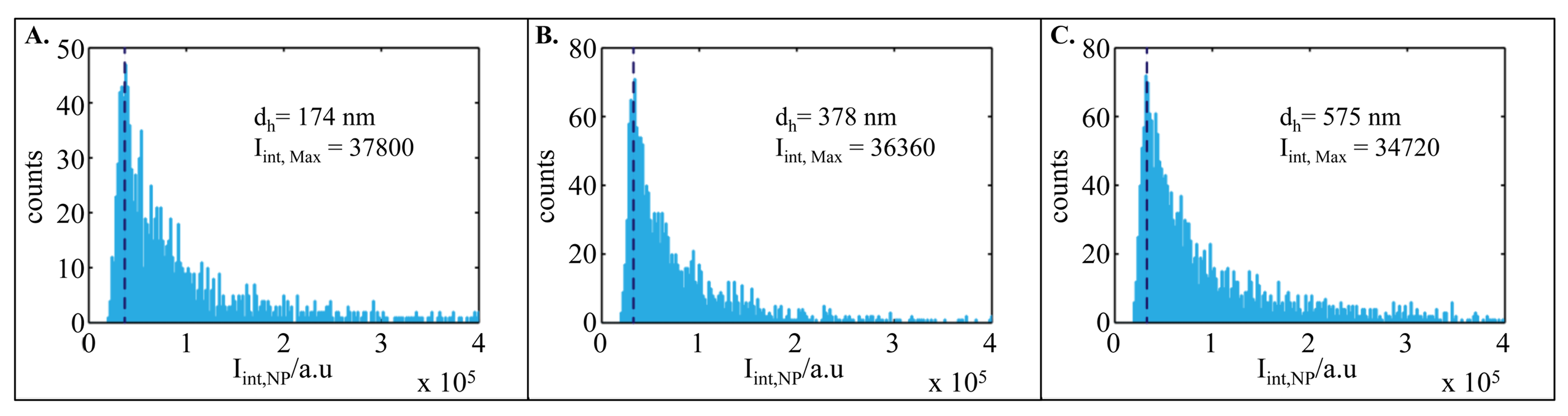

Supplement: Supplementary file 4 — Supporting Information [file BTM2-1-082-s004.tif]
