# Supplementary material for: Engineering of nanoparticle size via electrohydrodynamic jetting
Source: Bioeng Transl Med. 2016 Jun 20;1(1):82–93. doi: 10.1002/btm2.10010 (PMC5689507; doi:10.1002/btm2.10010)
Supplement: Supplementary file 5 — Supporting Information [file BTM2-1-082-s005.pdf]

Supplemental Information:

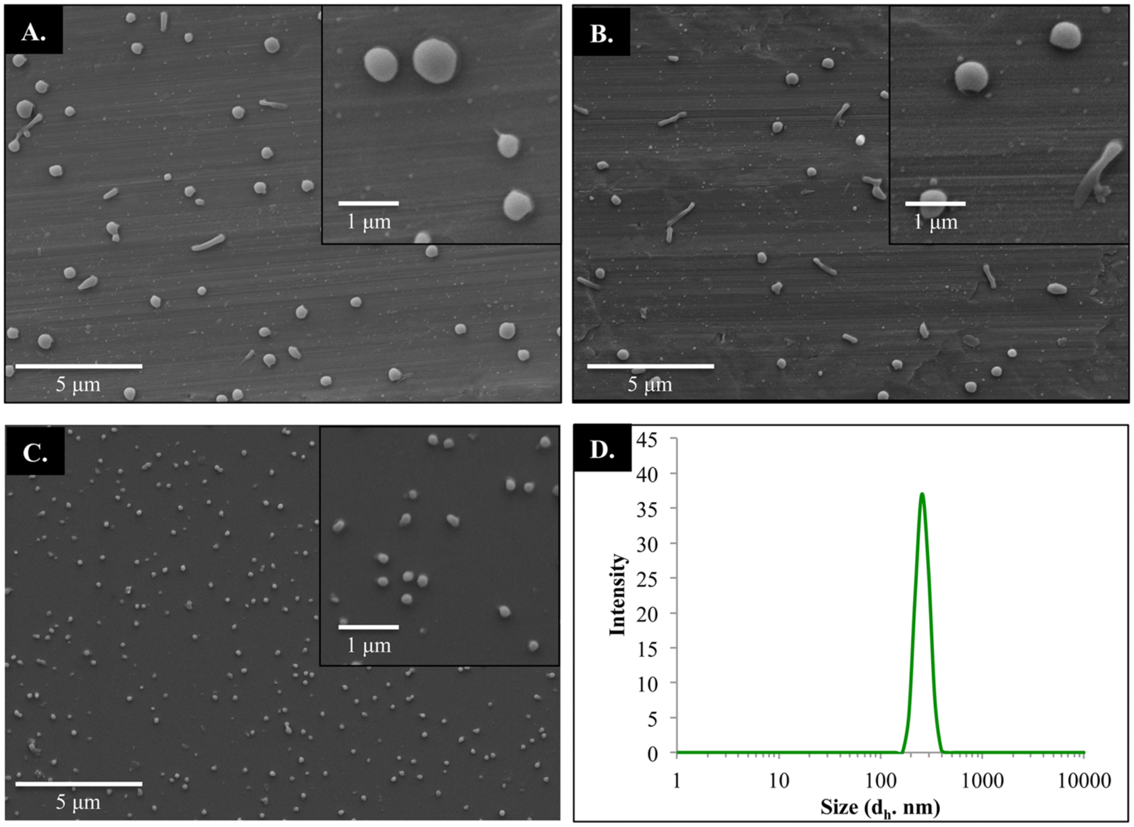

Supplemental Figure 1: PLGA nanoparticles fabricated through the EHD co-jetting technique. PLGA concentrations of 1, 0.5, and 0.03% w/v in DMF (A-C respectively) were sequentially tested to fabricate the smaller nanoparticle size ranges. (D) DLS measurement of the nanoparticles in PBS (C, at 0.03% w/v) displaying the average hydrodynamic diameter (d_h) of approximately 250 nm. This is larger than the observed size in the SEM images and is most likely due to partial aggregation of the nanoparticles.

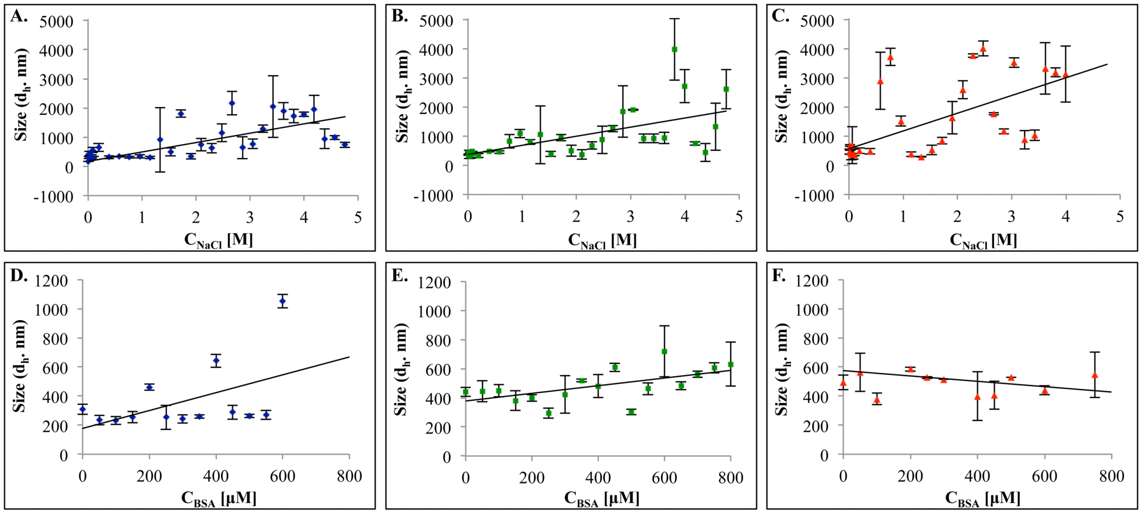

Supplemental Figure 2: Stability measurements of nanoparticles in increasing salt and serum conditions. Intensity distributions of the hydrodynamic diameters (d_h) of the nanoparticles as recorded in the presence of different concentrations of sodium chloride and bovine serum albumin, respectively. A) and D) nanoparticles with initial size of 174 nm, B) and E) nanoparticles with initial size of 378 nm, and C) and F) nanoparticles with initial size of 575 nm.

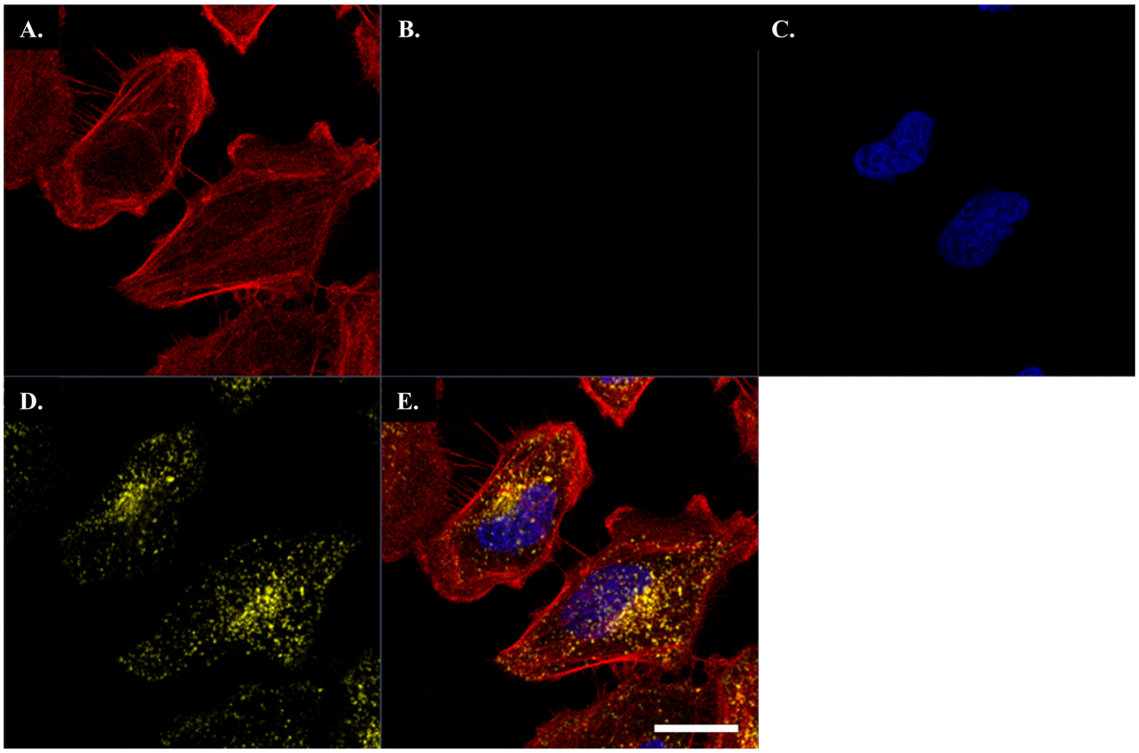

Supplemental Figure 3: A representative CLSM image of immunostained HeLa cells (control) without exposure to the nanoparticles after 12 hours of incubation. Stained cellular compartments are shown as A) cytoskeleton, B) green fluorescence channel in the absence of fluorescent nanoparticles, C) nuclei, D) lysosomes, and E) an overlay of all channels showing the above mentioned cellular compartments. The scale bar corresponds to 20 µm.

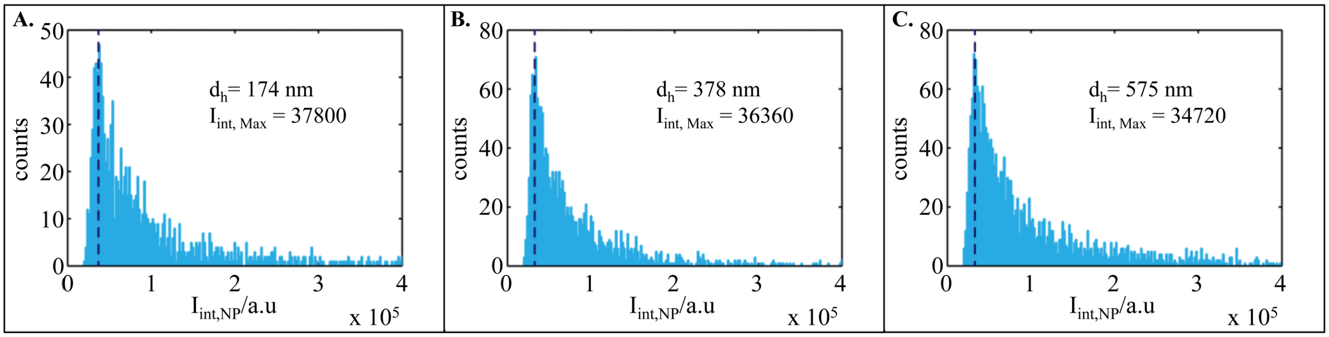

Supplemental Figure 4: Fluorescent intensity measurements of nanoparticles. Distribution of the integrated fluorescence intensities of nanoparticles $I_{Int,NP}$ among the different samples. The peak of the histogram was picked as reference $I_{Int,NP}$-value corresponding to one single nanoparticle. As the integrated fluorescence intensity per nanoparticle was scaled with the volume of the nanoparticles, different excitation power and detection gain was used for different kinds of nanoparticles resulting in more or less the same values for $I_{Int,NP}$ among the different nanoparticle species.
